# Remarks on the Washing out of the Stomach

**Published:** 1884-01

**Authors:** A. C. L. Ramsay

**Affiliations:** Chicago


### ARTICLE III.

**REMARKS ON THE WASHING OUT OF THE STOMACH. By Dr. A. C. L. RAMSAY, Chicago.**

Of this promising measure lately introduced as a therapeutical agent, little is generally known in this country.

The instruments generally used for the operation are—the pump of Kussmaul, the instrument of Dujardin-Beaumetz, the siphon-tube of Prof. Faucher, the ingenious instrument lately devised by Dr. Victor Andhoui of Paris.

For good and thorough work, and especially for hospitals, the last-named instrument is by far preferable, but for the average practitioner the siphon-tube will be found more adaptable for two reasons:

1st. The great facility with which it can be introduced, the patient being able to introduce the tube himself after a few sittings. 2d. Because it can be improvised almost everywhere, a piece of rubber tubing, four and one-half to five feet in length and of sufficient caliber, can be easily obtained, and every well regulated household has a common tin funnel which completes the instrumental outfit.

I will first describe the operation and then review the use of it in deseased conditions of the stomach.

The patient should be in the sitting position, the head looking directly forward at almost a right angle with the vertebral column. Prof. Constantine Paul prefers reversing the patient's head backwards in order to introduce the instrument vertically. Both methods are good, one succeeding better in some cases and vice versa. The spasmodic contractions of the pharyngeal muscles generally renders the first introduction very difficult. The application of a solution of bromide of potassium with a spray to the pharyngeal walls will be of great use in those cases. Then we introduce a common rigid œsophageal sound. After the second or third introduction, all spasms will be reduced to a minimum and the siphon-tube can be introduced simply by the patient going through the act of swallowing. A line drawn across the tube at eighteen or twenty inches from its free extremity will, when touching the lips, indicate that the tube has reached the fundus of the stomach, but this is generally useless as the rebellious action of the muscular coats of the stomach will always indicate that the tube is far enough.

It should be borne in mind that sometimes there is a great escape of gasses and often of the contents of the stomach, a hint well worth remembering, as both the patient and physician are liable to have soiled linen when it is overlooked. The tube should be well oiled and, it is needless to say, of smaller caliber for females. The tube being introduced, its free extremity is raised above the stomach as much as possible, and the funnel is applied to the end, water, preferably summer-cold, is then poured in, from one to two quarts, according to the capacity of the stomach. Then by bringing the funnel below the epigastrium the water can be seen filling the funnel and holding the contents of the stomach in suspension. This must be repeated two or three times using warm water for the last washing. The operation is generally concluded by introducing four or five ounces of warm milk in the stomach. The gaseous mineral waters are highly spoken of by the Germans in the washing out of the stomach, the sedative action of the carbonic acid gas, rendering the operation more pleasant to the patient. Dr. Andhoui reports wonderful results with the waters of Châtel-Guyon.

The Germans prefer alkaline waters, giving as their reason that the introduction of an alkaline fluid in an acid stomach is attended by the evolution of a certain amount of carbonic acid gas and acts as a local sedative.

It is a good plan after the operation to tie a large bandage snugly over the stomach, to prevent dilatation of the pyloric orifice, but where there is piloric stenosis this would seem to be a desirable result to obtain. It is well to have the patient walk about for a little while after each operation.

Twelve years ago Prof. Kussmaul first began advocating washing out of the stomach, with his pump, in what Chornel has so ably described in his work, "Des Dyspepsies," as the dyspepsia of liquids (dyspepsie des liquides), but which in reality is nothing but pure and simple dilatation of the stomach; and there is no disease where it will do more effective work than in the above. That is especially true in the dilatation caused by the gastritis of drinkers where the dilation seems to be due to a partial atrophy of the muscular coat of the stomach. The results are most wonderful, from a state of inanition the patient passes into one of improved nutrition and comfort.

The vomiting, which is such a constant symptom in those cases, disappears, and when the case is not accompanied by cirrhosis of the liver a speedy cure is effected, while when the unfortunate complication of cirrhosis is present, the patient is made quite comfortable and life is prolonged sometimes months when it was a question of weeks before the washing of the stomach was resorted to.

It is wonderful what dimensions the stomach will attain in cases of dilation, especially in habitual beer drinkers. I remember being present at a post mortem examination, held over the remains of an ex-saloon-keeper, where on dividing the abdominal walls in the mesial line, nothing but the enlarged stomach was to be seen. The intestines were crowded below and at the sides of it. Prof. Constantine Paul is authority for the statement that a patient at the Hotel Dieu of Paris had such an enormously distended stomach that it would hold forty-nine liters of water. The best time for the operation in those cases is at bedtime, when it will be found to prevent the usual vomiting the next morning.

Its use in cases of poisoning need not be commented upon. In obstinate cases of vomiting of pregnancy, where, as Prof. Delaskie Miller so ably pointed out, the excessive acidity of the secretions of the stomach is the greatest obstacle to overcome, I think the measure worthy of a trial.

